# Supplementary material for: Prevalence and uptake of vaping among people who have quit smoking: a population study in England, 2013-2024
Source: BMC Med. 2024 Nov 21;22:503. doi: 10.1186/s12916-024-03723-2 (PMC11580220; doi:10.1186/s12916-024-03723-2)
Supplement: Supplementary file 1 — Additional file 1: TableS1. Modelled estimates of the prevalence of current vaping and late uptake of vaping after smoking cessation among subgroups of ex-smokers in England, in the first and last months of the study period. [file 12916_2024_3723_MOESM1_ESM.pdf]

Table S1. Modelled estimates of the prevalence of current vaping and late uptake of vaping after smoking cessation among subgroups of ex-smokers in England, in the first and last months of the study period

	Prevalence, % [95%CI][1]			
	Current vaping[2]		Late uptake after smoking cessation[3]	
	Oct 2013	May 2024	Oct 2013	May 2024
Year of age[4]				
18	4.5 [2.1–9.2]	58.9 [50.3–67.0]	-	-
25	4.2 [2.5–7.1]	50.4 [44.1–56.7]	-	-
35	3.9 [2.8–5.2]	38.3 [34.8–41.8]	0.7 [0.3–2.0]	10.1 [4.9–19.7]
45	3.3 [2.4–4.4]	27.2 [24.5–30.0]	0.6 [0.3–1.3]	7.4 [5.2–10.5]
55	2.3 [1.6–3.3]	17.8 [15.6–20.4]	0.5 [0.2–1.3]	5.2 [3.6–7.4]
65	1.2 [0.8–1.8]	10.7 [9.1–12.5]	0.3 [0.1–0.8]	3.4 [2.2–5.3]
Gender				
Men	2.0 [1.4–2.7]	21.2 [18.8–23.7]	0.4 [0.2–1.0]	4.7 [3.3–6.7]
Women	1.9 [1.4–2.7]	19.3 [17.0–21.9]	0.4 [0.2–1.0]	2.7 [1.7–4.3]
Occupational social grade				
ABC1 (more advantaged)	1.4 [1.0–2.1]	17.7 [15.8–19.7]	0.3 [0.1–0.7]	2.7 [1.8–3.8]
C2DE (less advantaged)	2.8 [2.0–3.9]	23.8 [21.0–27.0]	0.6 [0.2–1.4]	5.2 [3.5–7.8]
	Current vaping[2]		Late uptake after smoking cessation[3]	
	Apr 2014	May 2024	Apr 2014	May 2024
Level of alcohol consumption (AUDIT-C score)[5]				
0 (lowest)	3.8 [2.2–6.4]	21.1 [18.0–24.7]	0.7 [0.1–4.8]	4.2 [2.5–7.2]
3	2.2 [1.5–3.2]	18.2 [16.1–20.5]	0.5 [0.2–1.6]	2.5 [1.8–3.7]
6	1.8 [1.1–3.0]	19.9 [17.6–22.5]	0.5 [0.1–1.8]	3.0 [2.0–4.6]
9	2.2 [1.1–4.4]	26.6 [22.2–31.4]	0.5 [0.1–2.6]	6.3 [3.8–10.4]
12 (highest)	2.7 [0.8–9.4]	35.4 [25.9–46.3]	0.5 [0.0–8.6]	13.9 [5.7–30.1]

[1] Data are weighted estimates of prevalence in the first and last months in the study period from logistic regression with survey month modelled non-linearly using restricted cubic splines (five knots).
[2] Current vaping among ≥1y ex-smokers.
[3] Current vaping among ex-smokers who quit smoking before e-cigarettes became popular in 2011.
[4] Modelled estimates for selected ages. Note that the model used to derive these estimates included data from participants of all ages (≥18y), not only those who were aged exactly 18, 25, 35, 45, 55, or 65 years. We do not report estimates of the prevalence of late uptake of vaping after smoking cessation among 18- and 25-year-olds because very few participants in this age range could have quit smoking as an adult before 2011.
[5] AUDIT-C scores range from 0 to 12. Note that the model used to derive these estimates included data from participants with any score on this scale, not only those with a score of exactly 0, 3, 6, 9, or 12. AUDIT-C data first collected April 2014.
