# Supplementary material for: Prevalence and uptake of vaping among people who have quit smoking: a population study in England, 2013-2024
Source: BMC Med. 2024 Nov 21;22:503. doi: 10.1186/s12916-024-03723-2 (PMC11580220; doi:10.1186/s12916-024-03723-2)
Supplement: Supplementary file 2 — Additional file 2: FigS1. Trend in late uptake of vaping after smoking cessation (≥14y ex-smokers), October 2013 to May 2024. [file 12916_2024_3723_MOESM2_ESM.pdf]

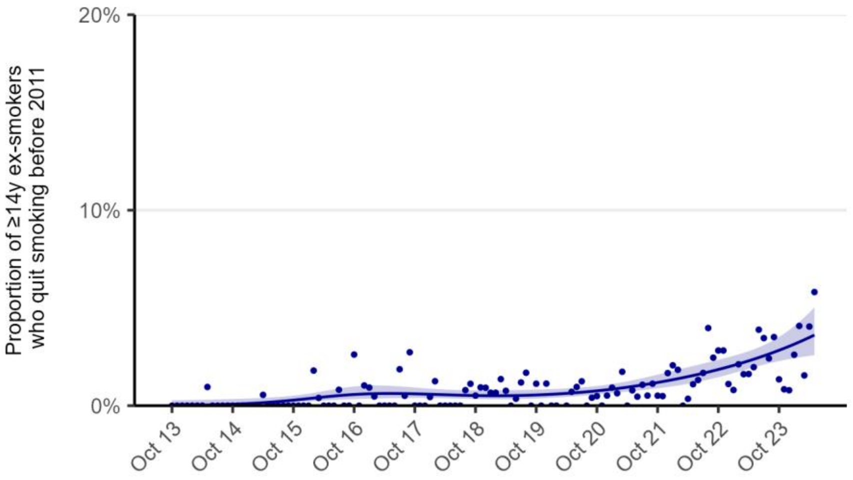

Figure S1. Trend in late uptake of vaping after smoking cessation (≥14y ex-smokers), October 2013 to May 2024.

Prevalence of current vaping among ≥14y ex-smokers who quit smoking before e-cigarettes started to become popular in 2011 (*n*=23,740). Line represents the modelled weighted proportion by monthly survey wave (modelled non-linearly using restricted cubic splines with five knots). Shaded band represents 95% confidence intervals. Points represent the unmodelled weighted proportion by month.
