# Supplementary material for: Prevalence and uptake of vaping among people who have quit smoking: a population study in England, 2013-2024
Source: BMC Med. 2024 Nov 21;22:503. doi: 10.1186/s12916-024-03723-2 (PMC11580220; doi:10.1186/s12916-024-03723-2)
Supplement: Supplementary file 3 — Additional file 3. Results for changes in the profile of ex-smokers who vape; Tables S2-S3 and Figure S2. TableS2. Changes in the profile of ex-smokers who vape since disposable e-cigarettes started to become popular. TableS3. Changes in the profile of ex-smokers who vape since disposable e-cigarettes started to become popular (sensitivity analysis restricted to data from April 2020 onwards). FigS2. Trend in the mean duration of abstinence from smoking by survey year among ex-smokers who vape, October 2013 to May 2024. [file 12916_2024_3723_MOESM3_ESM.pdf]

Additional File 3

Changes in the profile of ex-smokers who vape

There were several differences in the profile of ≥1y ex-smokers who vaped from before (July 2016 to May 2021) to after (June 2021 to May 2024) disposable e-cigarettes started to become popular (**Table S2**).

Ex-smoking vapers surveyed since disposables started to become popular reported a greater duration of abstinence from smoking, on average, than those surveyed earlier (6.2 vs. 4.7y). Unplanned analyses showed the mean duration of abstinence among ex-smoking vapers increased non-linearly across the entire study period, with modelled estimates increasing from 2.1y [1.6–2.5y] in 2013/14 to 5.6y [5.3–5.9y] in 2019/20 and then appearing to level off (reaching 6.2y [5.8–6.7y] in 2023/24; **Figure S2**). In addition, a greater proportion were aged 18-34y (34.6% vs. 27.3%). There were no notable changes in terms of gender, occupational social grade, or alcohol consumption.

In terms of vaping characteristics, ex-smoking vapers surveyed since disposables started to become popular were more likely than those surveyed earlier to report having been vaping for more than a year (91.6% vs. 87.2%) and less likely to report having been vaping for <1 week (0.2% vs. 1.2%) or for between 6 months and a year (4.6% vs. 7.0%). They were also more likely to say they did not know how frequently they vaped (18.7% vs. 12.7%) and less likely to report vaping daily (72.8% vs. 79.6%). They were much more likely to report mainly using disposable e-cigarettes (16.7% vs. 2.0%), offset by a decline in the use of refillable devices (70.9% vs. 85.1%). They were more likely to report using the highest-strength (≥20 mg/ml) nicotine e-liquids (11.8% vs. 3.0%) or to say that they did not know the strength of their nicotine-containing e-liquid (4.1% vs. 2.2%). They were also more likely to report buying their vaping products from supermarkets and convenience stores (32.0% vs. 26.8%) and less likely to report buying them from vape shops (28.7% vs. 35.2%) or 'other' sources (4.4% vs. 7.2%).

A sensitivity analysis restricted to participants who were interviewed via telephone (April 2020 onwards; **Table S3**) showed a broadly similar pattern. However, there were two notable differences. First, when the pre-disposables period was shortened (starting at April 2020 rather than July 2016), vaping duration was more similar across ex-smoking vapers surveyed after compared with before disposables started to become popular (e.g., 91.6% vs. 93.5% reported having vaped for more than a year). Second, in addition to an increase in the proportion of ex-smoking vapers who reported mainly buying their products from supermarkets and convenience stores since disposables started to become popular (32.0% vs. 26.5%), the proportion who reported mainly buying from vape shops

increased (28.7% vs. 23.4%) and the proportion mainly buying from online sources decreased (34.9% vs. 42.6%).

Table S2. Changes in the profile of ex-smokers who vape since disposable e-cigarettes started to become popular

	≥1y ex-smokers who currently vape		
	**Pre-disposables period[1], %[2] [95%CI]**	**Disposables period[1], %[2] [95%CI]**	**Δ [95% CI][3]**
Unweighted N	1,578	1,821	-
**Quitting history**			
Duration of abstinence from smoking, mean [95%CI] years	4.7 [4.4–5.0]	6.2 [5.9–6.5]	1.5 [1.1; 1.9]
**Sociodemographic profile**			
Age, years			
Mean [95%CI]	45.5 [44.8–46.2]	43.3 [42.6–44.0]	-2.2 [-3.2; -1.2]
18-24	5.4 [4.2–6.6]	9.4 [7.9–10.9]	4.0 [2.0; 5.9]
25-34	21.9 [19.7–24.2]	25.2 [23.0–27.5]	3.3 [0.1; 6.5]
35-44	22.1 [19.8–24.4]	23.2 [21.1–25.4]	1.1 [-2.0; 4.3]
45-54	23.0 [20.7–25.2]	19.0 [17.1–20.9]	-4.0 [-6.9; -1.1]
55-64	16.7 [14.9–18.6]	13.2 [11.7–14.8]	-3.5 [-5.9; -1.1]
≥65	10.8 [9.4–12.3]	10.0 [8.5–11.4]	-0.9 [-2.9; 1.2]
Women	43.5 [40.8–46.1]	46.8 [44.3–49.3]	3.3 [-0.3; 6.9]
Social grade C2DE (less advantaged)	49.5 [46.8–52.1]	51.6 [49.1–54.1]	2.1 [-1.5; 5.8]
**Drinking profile**			
Level of alcohol consumption (AUDIT-C score)			
Mean [95%CI]	4.0 [3.8–4.2]	3.9 [3.8–4.1]	-0.1 [-0.3; 0.2]
0 (non-drinker)	24.0 [21.7–26.3]	22.5 [20.3–24.6]	-1.5 [-4.6; 1.6]
1-4 (low-risk)	33.0 [30.4–35.5]	36.0 [33.5–38.4]	3.0 [-0.5; 6.5]
5-12 (increasing/higher-risk)	43.0 [40.4–45.7]	41.5 [39.0–44.0]	-1.5 [-5.2; 2.1]
**Vaping profile**			
Vaping duration (weeks)			
<1	1.2 [0.6–1.9]	0.2 [0.0–0.6]	-1.0 [-1.7; -0.3]
1-6	0.9 [0.4–1.4]	0.7 [0.0–1.4]	-0.2 [-1.1; 0.7]
>6-12	1.5 [0.9–2.1]	0.9 [0.3–1.5]	-0.6 [-1.5; 0.3]
>12-26	1.7 [1–2.4]	1.3 [0.5–2.1]	-0.4 [-1.5; 0.6]
>26-52	7.0 [5.6–8.3]	4.6 [3.1–6.1]	-2.4 [-4.4; -0.4]
>52	87.2 [85.4–89.0]	91.6 [89.6–93.6]	4.4 [1.7; 7.1]
Don't know	0.5 [0.1–0.8]	0.7 [0.1–1.3]	0.2 [-0.5; 0.9]

Table continues on next page.

Table S2. *continued*

	≥1y ex-smokers who currently vape		
	**Pre-disposables period[1], %[2] [95%CI]**	**Disposables period[1], %[2] [95%CI]**	**Δ [95% CI][3]**
Vaping frequency			
Non-daily	7.7 [6.3–9.1]	8.5 [6.5–10.6]	0.9 [-1.6; 3.3]
Less than once a week	2.5 [1.7–3.3]	2.5 [1.4–3.6]	0.0 [-1.4; 1.4]
Not every day but at least once a week	5.2 [4.0–6.3]	6.0 [4.3–7.8]	0.9 [-1.2; 3.0]
Daily	79.6 [77.4–81.8]	72.8 [69.6–76.1]	-6.8 [-10.7; -2.9]
Once a day	3.4 [2.4–4.4]	5.1 [3.6–6.7]	1.7 [-0.2; 3.6]
Twice a day	2.8 [1.9–3.7]	3.5 [2.1–5.0]	0.7 [-1.0; 2.4]
3-4 times a day	5.9 [4.6–7.2]	5.8 [4.1–7.5]	-0.1 [-2.3; 2.0]
5-7 times a day	10.9 [9.2–12.5]	9.9 [7.8–12.1]	-0.9 [-3.7; 1.8]
8-11 times a day	16.7 [14.6–18.7]	13.7 [11.2–16.2]	-3.0 [-6.2; 0.3]
≥12 times a day	40.0 [37.3–42.6]	34.7 [31.2–38.3]	-5.2 [-9.6; -0.8]
Don't know	12.7 [10.8–14.6]	18.7 [15.8–21.5]	5.9 [2.6; 9.3]
Main device type			
Refillable	85.1 [83.2–87.1]	70.9 [68.4–73.3]	-14.3 [-17.4; -11.2]
Disposable	2.0 [1.2–2.7]	16.7 [14.7–18.8]	14.8 [12.6; 16.9]
Pod	12.5 [10.7–14.2]	11.4 [9.7–13.0]	-1.1 [-3.5; 1.3]
Don't know	0.4 [0.1–0.8]	1.0 [0.4–1.6]	0.6 [-0.1; 1.3]
Nicotine strength			
No nicotine	11.6 [9.9–13.3]	11.3 [9.0–13.6]	-0.3 [-3.1; 2.5]
>0 to 6 mg/ml	45.1 [42.3–47.8]	40.1 [36.4–43.7]	-5.0 [-9.5; -0.5]
7-11 mg/ml	9.9 [8.3–11.5]	11.5 [9.1–14.0]	1.6 [-1.3; 4.6]
12-19 mg/ml	28.1 [25.6–30.5]	20.4 [17.4–23.4]	-7.7 [-11.6; -3.8]
20 mg/ml or more[6]	3.0 [2.1–3.8]	11.8 [9.4–14.2]	8.9 [6.3; 11.4]
Don't know if it contains nicotine	0.3 [0.0–0.5]	0.8 [0.1–1.5]	0.6 [-0.2; 1.3]
Contains nicotine but don't know the strength	2.2 [1.4–2.9]	4.1 [2.6–5.6]	1.9 [0.3; 3.6]
Source of purchase			
Vape shop	35.2 [32.6–37.8]	28.7 [25.3–32.1]	-6.5 [-10.8; -2.2]
Supermarket/convenience store	26.8 [24.4–29.1]	32.0 [28.5–35.4]	5.2 [1.0; 9.4]
Online	30.9 [28.4–33.4]	34.9 [31.4–38.4]	4.0 [-0.3; 8.3]
Other	7.2 [5.7–8.6]	4.4 [2.9–5.9]	-2.8 [-4.8; -0.7]

[1] Pre-disposables period: July 2016 – May 2021. Disposables period: June 2021 – May 2024. Results of a sensitivity analysis in which we restricted the sample to those surveyed via the same modality (telephone: from April 2020 onwards) are shown in **Table S3**.
[2] Column percentages.
[3] Absolute percentage point change from the pre-disposables to disposables period.

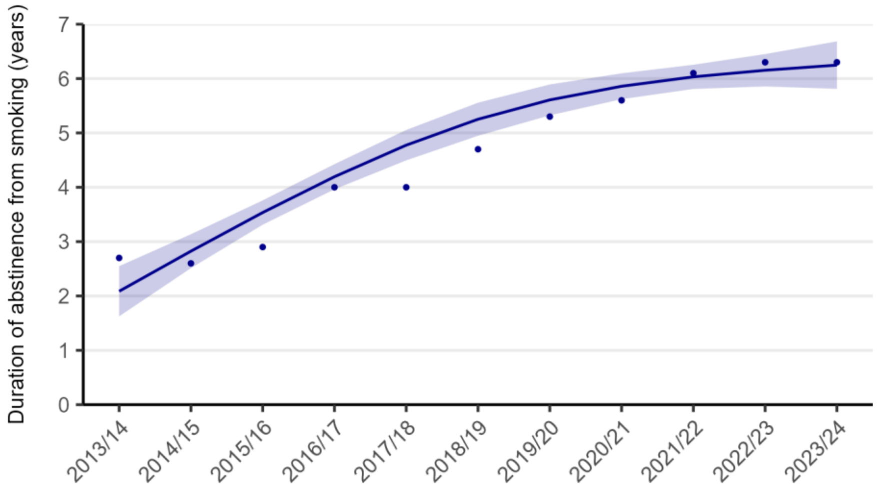

Figure S2. Trend in the mean duration of abstinence from smoking by survey year among ex-smokers who vape, October 2013 to May 2024.

Mean duration of abstinence among ≥1y ex-smokers who vape. Line represents the modelled weighted proportion by survey year (12-month periods from October to the following September [e.g., 2013/14 = October 2013 to September 2014, etc.]; modelled non-linearly using restricted cubic splines with three knots). Shaded band represents 95% confidence intervals. Points represent the unmodelled weighted proportion by year.

Additional File 3

Table S3. Changes in the profile of ex-smokers who vape since disposable e-cigarettes started to become popular (sensitivity analysis restricted to data from April 2020 onwards)

	≥1y ex-smokers who currently vape		
	**Pre-disposables period[1], %[2] [95%CI]**	**Disposables period[1], %[2] [95%CI]**	**Δ [95% CI][3]**
Unweighted N	529	1,821	-
**Quitting history**			
Duration of abstinence from smoking, mean (SD) years	5.3 [5.0–5.7]	6.2 [5.9–6.5]	0.9 [0.4; 1.3]
**Sociodemographic profile**			
Age, years			
Mean (SD)	45.1 [43.8–46.4]	43.3 [42.6–44]	-1.8 [-3.3; -0.3]
18-24	6.4 [4.0–8.7]	9.4 [7.9–10.9]	3.0 [0.2; 5.9]
25-34	22.4 [18.5–26.3]	25.2 [23.0–27.5]	2.8 [-1.7; 7.3]
35-44	23.6 [19.5–27.7]	23.2 [21.1–25.4]	-0.4 [-5.0; 4.3]
45-54	21.2 [17.5–24.8]	19.0 [17.1–20.9]	-2.2 [-6.3; 1.9]
55-64	15.5 [12.4–18.6]	13.2 [11.7–14.8]	-2.3 [-5.7; 1.2]
≥65	11.0 [8.4–13.6]	10.0 [8.5–11.4]	-1.0 [-4.0; 1.9]
Women	43.6 [39.1–48.1]	46.8 [44.3–49.3]	3.2 [-1.9; 8.3]
Social grade C2DE (less advantaged)	49.9 [45.3–54.4]	51.6 [49.1–54.1]	1.7 [-3.5; 6.9]
**Drinking profile**			
Level of alcohol consumption (AUDIT-C score)			
Mean (SD)	4.0 [3.7–4.3]	3.9 [3.8–4.1]	-0.1 [-0.4; 0.3]
0 (non-drinker)	21.9 [18.0–25.8]	22.5 [20.3–24.6]	0.6 [-3.9; 5.0]
1-4 (low-risk)	34.4 [29.9–38.9]	36.0 [33.5–38.4]	1.6 [-3.5; 6.7]
5-12 (increasing/higher-risk)	43.7 [39.1–48.3]	41.5 [39.0–44.0]	-2.2 [-7.4; 3.1]
**Vaping profile**			
Vaping duration (weeks)			
<1	0	0.2 [0.0–0.6]	0.2 [-0.1; 0.6]
1-6	0	0.7 [0.0–1.4]	0.7 [0.0; 1.4]
>6-12	0.2 [0.0–0.5]	0.9 [0.3–1.5]	0.7 [0.1; 1.4]
>12-26	1.4 [0.3–2.5]	1.3 [0.5–2.1]	-0.1 [-1.4; 1.2]
>26-52	4.7 [2.8–6.6]	4.6 [3.1–6.1]	-0.1 [-2.5; 2.3]
>52	93.5 [91.3–95.7]	91.6 [89.6–93.6]	-1.9 [-4.9; 1.1]
Don't know	0.3 [0.0–0.7]	0.7 [0.1–1.3]	0.4 [-0.3; 1.1]

Table continues on next page.

Additional File 3

Table S3. *continued*

	≥1y ex-smokers who currently vape		
	**Pre-disposables period[1], %[2] [95%CI]**	**Disposables period[1], %[2] [95%CI]**	**Δ [95% CI][3]**
Vaping frequency			
Non-daily	9.3 [6.6–12.1]	8.5 [6.5–10.6]	-0.8 [-4.2; 2.6]
Less than once a week	2.4 [1.0–3.7]	2.5 [1.4–3.6]	0.1 [-1.6; 1.9]
Not every day but at least once a week	7.0 [4.5–9.4]	6.0 [4.3–7.8]	-0.9 [-3.9; 2.0]
Daily	73.9 [69.7–78.1]	72.8 [69.6–76.1]	-1.1 [-6.3; 4.2]
Once a day	3.7 [1.7–5.6]	5.1 [3.6–6.7]	1.5 [-1.0; 4.0]
Twice a day	4.1 [2.1–6.2]	3.5 [2.1–5.0]	-0.6 [-3.1; 1.9]
3-4 times a day	6.0 [3.7–8.3]	5.8 [4.1–7.5]	-0.2 [-3.1; 2.7]
5-7 times a day	9.3 [6.6–12.0]	9.9 [7.8–12.1]	0.7 [-2.8; 4.1]
8-11 times a day	15.5 [12.0–18.9]	13.7 [11.2–16.2]	-1.8 [-6.0; 2.5]
≥12 times a day	35.4 [30.8–40.0]	34.7 [31.2–38.3]	-0.6 [-6.4; 5.1]
Don't know	16.8 [13.2–20.3]	18.7 [15.8–21.5]	1.9 [-2.6; 6.4]
Main device type			
Refillable	87.7 [84.6–90.7]	70.9 [68.4–73.3]	-16.8 [-20.7; -12.9]
Disposable	1.0 [0.1–1.8]	16.7 [14.7–18.8]	15.8 [13.6; 18.0]
Pod	11.0 [8.0–13.9]	11.4 [9.7–13.0]	0.4 [-3.0; 3.8]
Don't know	0.4 [0.0–0.8]	1.0 [0.4–1.6]	0.6 [-0.1; 1.4]
Nicotine strength			
No nicotine	12.8 [9.6–16.1]	11.3 [9.0–13.6]	-1.5 [-5.5; 2.4]
>0 to 6 mg/ml	47.7 [42.9–52.6]	40.1 [36.4–43.7]	-7.7 [-13.7; -1.7]
7-11 mg/ml	7.5 [5.1–10.0]	11.5 [9.1–14.0]	4.0 [0.5; 7.5]
12-19 mg/ml	25.9 [21.7–30.0]	20.4 [17.4–23.4]	-5.5 [-10.6; -0.4]
20 mg/ml or more[6]	1.6 [0.4–2.7]	11.8 [9.4–14.2]	10.2 [7.6; 12.9]
Don't know if it contains nicotine	0.5 [0.0–1.0]	0.8 [0.1–1.5]	0.3 [-0.5; 1.2]
Contains nicotine but don't know the strength	4.0 [2.2–5.8]	4.1 [2.6–5.6]	0.1 [-2.3; 2.4]
Source of purchase			
Vape shop	23.4 [19.4–27.4]	28.7 [25.3–32.1]	5.3 [0.0; 10.5]
Supermarket/convenience store	26.5 [22.3–30.7]	32.0 [28.5–35.4]	5.5 [0.0; 10.9]
Online	42.6 [37.8–47.4]	34.9 [31.4–38.4]	-7.6 [-13.6; -1.7]
Other	7.5 [4.8–10.3]	4.4 [2.9–5.9]	-3.1 [-6.3; 0.0]

[1] Pre-disposables period: Apr 2020 – May 2021. Disposables period: June 2021 – May 2024.

[2] Column percentages.

[3] Absolute percentage point change from the pre-disposables to disposables period.
